# Path integral Monte Carlo approach to the structural properties and collective excitations of liquid $^3{\text {He}}$ without fixed nodes

**DOI:** 10.1038/s41598-021-04355-9

**Published:** 2022-01-13

**Authors:** Tobias Dornheim, Zhandos A. Moldabekov, Jan Vorberger, Burkhard Militzer

**Affiliations:** 1 Center for Advanced Systems Understanding (CASUS), 02826 Görlitz, Germany; 2 Helmholtz-Zentrum Dresden-Rossendorf (HZDR), 01328 Dresden, Germany; 3 Department of Earth and Planetary Science, University of California, Berkeley, CA 94720 USA; 4 Department of Astronomy, University of California, Berkeley, CA 94720 USA

**Keywords:** Chemical physics, Atomic and molecular physics

## Abstract

Due to its nature as a strongly correlated quantum liquid, ultracold helium is characterized by the nontrivial interplay of different physical effects. Bosonic $^4{\text {He}}$ exhibits superfluidity and Bose-Einstein condensation. Its physical properties have been accurately determined on the basis of *ab initio* path integral Monte Carlo (PIMC) simulations. In contrast, the corresponding theoretical description of fermionic $^3{\text {He}}$ is severely hampered by the notorious fermion sign problem, and previous PIMC results have been derived by introducing the uncontrolled fixed-node approximation. In this work, we present extensive new PIMC simulations of normal liquid $^3{\text {He}}$ without any nodal constraints. This allows us to to unambiguously quantify the impact of Fermi statistics and to study the effects of temperature on different physical properties like the static structure factor $S({\mathbf {q}})$, the momentum distribution $n({\mathbf {q}})$, and the static density response function $\chi ({\mathbf {q}})$. In addition, the dynamic structure factor $S({\mathbf {q}},\omega )$ is rigorously reconstructed from imaginary-time PIMC data. From simulations of $^3{\text {He}}$, we derived the familiar phonon–maxon–roton dispersion function that is well-known for $^4{\text {He}}$ and has been reported previously for two-dimensional $^3{\text {He}}$ films (Nature 483:576–579 (2012)). The comparison of our new results for both $S({\mathbf {q}})$ and $S({\mathbf {q}},\omega )$ with neutron scattering measurements reveals an excellent agreement between theory and experiment.

## Introduction

Ultracold helium constitutes one of the most actively investigated quantum systems and has been of central relevance for our understanding of important physical concepts such as superfluidity^1^ and Bose–Einstein condensation^2^. Due to its nature as a strongly correlated quantum liquid, helium exhibits an intricate interplay of non-ideality effects, quantum statistics, and thermal excitations. Naturally, an accurate description of physical effects such as the lambda phase transition of $^4{\text {He}}$ must capture all of these effects simultaneously—a challenging task beyond simple mean-field models and perturbative approaches.

This challenge was met by Feynman^3^ in terms of the path integral formalism that exactly maps the interacting quantum system of interest onto an effective classical system of ring polymers^4^. Specifically, this quest for an accurate description of helium^5^ has given rise to the widely used path integral Monte Carlo (PIMC) simulation method^6–8^, one of the most successful tools in statistical physics, quantum chemistry, and related disciplines (We note that Monte Carlo methods in general are applied in a gamut of different contexts, like solid state physics^9,10^ or the investigation of magnetic properties^11–15^). Recently the *worm algorithm*^16,17^ elegantly addressed the challenge of sampling the permutation space ergodically. This is required to take into account the effect of quantum statistics, which is nontrivial because of the strong short-range repulsion between two He atoms.

The PIMC method gives straightforward access to important physical observables like the superfluid fraction^18^, the momentum distribution^1^, and the static structure factor, which has resulted in excellent agreement between theory and experiments for $^4{\text {He}}$; see the review by Ceperley^1^ for details. In addition, PIMC simulations can be used as the starting point for an analytic continuation^19^ that provides access to the dynamic structure factor $S({\mathbf {q}},\omega )$^20–22^—a key quantity in neutron scattering experiments^23–26^. In particular, PIMC-based data for $S({\mathbf {q}},\omega )$ have given important insights into the connection between superfluidity and roton-like quasi-particle excitations.

In stark contrast, PIMC simulations of $^3{\text {He}}$ are substantially hampered by the notorious fermion sign problem^27–29^, which leads to an exponential increase of the computation time with increasing the system size *N* or decreasing the temperature *T*. Therefore, Ceperley has introduced the uncontrolled *fixed-node approximation* into fermionic PIMC simulations^27^ and derived the first results for $^3{\text {He}}$. This investigation was restricted to the total energy, and the agreement to experimental data^30^ was inconclusive. Few PIMC studies of $^3{\text {He}}$ have been published since^31^. To our knowledge, no data have been presented for either the structural properties or the spectrum of collective excitations.

This is unfortunate, as ultracold $^3{\text {He}}$ offers a potential wealth of interesting physical effects such as a superfluid phase transition at $T\lesssim 2.5\;{\text {mK}}$^32^ that is triggered by the formation of Cooper pairs. Furthermore, it has been recently demonstrated^33,34^ that two-dimensional $^3\text {He}$ exhibits a rich phonon–maxon–roton dispersion relation that phenomenologically resembles the well-known dispersion of $^4{\text {He}}$. We also note that bulk $^3{\text {He}}$ is notoriously difficult to study with laboratory experiments^35^. A thorough theoretical approach is thus indispensable to understand the underlying physical mechanisms. Furthermore, progress has been made in characterizing $^3{\text {He}}$–$^4{\text {He}}$ mixtures with PIMC simulations^36–38^ but some disagreements regarding the kinetic energy remain between theoretical and experimental results.

**Figure 1:**
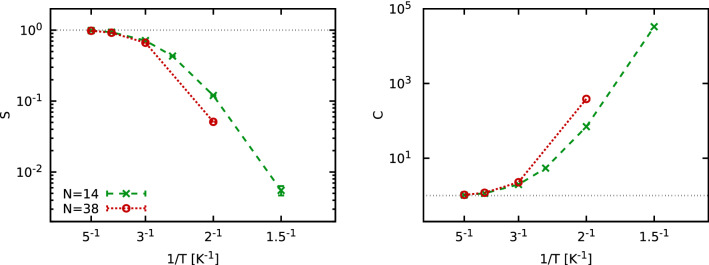
Average sign, *S*, (left) and increase in computational cost, $C=1/S^2$, (right) for $N=14$ and $N=38$ $^3{\text {He}}$ atoms as a function of the inverse temperature, $T^{-1}$.

In this work, we remedy this unsatisfactory situation by carrying out extensive direct PIMC simulations of normal liquid $^3{\text {He}}$ *without any nodal restrictions*. Therefore, our simulations are free of uncontrolled approximations, but computationally extremely costly when the temperature is decreased, cf. the discussion of Fig. 1 below. This allows us to present highly accurate results for the temperature dependence of important properties such as the static structure factor $S({\mathbf {q}})$, the momentum distribution function $n({\mathbf {q}})$, and the static density response function $\chi ({\mathbf {q}})$. Furthermore, we are able to unambiguously characterize the impact of Fermi statistics, which is comparably small for $S({\mathbf {q}})$ and $\chi ({\mathbf {q}})$, but very pronounced on $n({\mathbf {q}})$ in the small-momentum range.

We compute the imaginary time density–density correlation function $F({\mathbf {q}},\tau )$ for the same parameters, which gives us access to the dynamic structure factor $S({\mathbf {q}},\omega )$. First and foremost, we indeed find the familiar phonon–maxon–roton dispersion relation^33^ in these spectra, which is qualitatively similar to normal liquid $^4{\text {He}}$^22^ at comparable conditions. Our new PIMC data for the spectrum of collective excitations are in excellent agreement with results from neutron scattering experiments^24^. If they are available, experimental and theoretical results are found to be in excellent agreement.

To our knowledge, this constitutes the first comprehensive PIMC study of an ultracold atomic bulk system of fermions at finite temperature without the fixed-node approximation^27^, thereby opening up new avenues for the investigation of other applications such as quantum-dipole systems^39,40^, bilayer structures^41,42^, or isotopic mixtures of helium^36–38^.

## Results

We consider spin-unpolarized $^3{\text {He}}$ at a number density $n=N/V=0.016355$ Å$^{-3}$. Convergence with the number of imaginary-time steps has been carefully checked; see the “Methods” section for additional details. We note that the spin-polarization has negligible effects on the properties of $^3{\text {He}}$ at these conditions; see also the discussion of Fig. 3 below.

We begin our investigation by analyzing the fermion sign problem, which constitutes the main computational bottleneck. In Fig. 1, we show our PIMC results for the average sign *S* (see e.g. Ref.^29^) for $N=14$ and $N=38$ atoms interacting via the Aziz-2 potential^43^. *S* constitutes a measure for the amount of cancellation of positive and negative terms in the simulations. It monotonically decreases with *T*. In the high-temperature limit, *S* approaches 1 because the effects of quantum statistics vanish, whereas $S\rightarrow 0$ as the system approaches the ground state^44^. Furthermore, the error bar of an observable ${{\hat{A}}}$ scales as $\Delta A/A\sim 1/S$, resulting in an increase in computational cost of $C=1/S^2$^29^. This trend is shown in the right panel of Fig. 1 and can be interpreted as follows: For $T=5\;{\text {K}}$, which is close to the Fermi temperature of $^3{\text {He}}$, the effect of quantum statistics is negligible and there is no increase in computational cost, i.e., $C\sim 1$. In contrast, we find $C\sim 10^3$ for $T=2\;{\text {K}}$ and $N=38$, which means we need 1000 times as much computer time for the fermionic calculations as we do for the bosonic case without a sign problem. While this is still feasible with ${\mathcal {O}}(10^5)$ CPU hours, it constitutes the limit of the present investigation.

**Figure 2:**
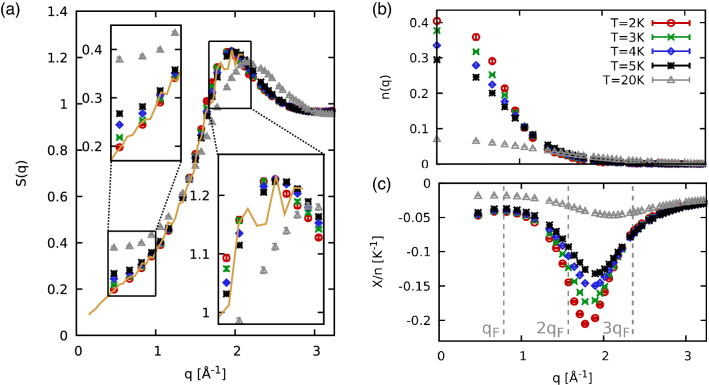
Static properties of $^3{\text {He}}$ for different temperatures *T*: Shown are PIMC results for the static structure factor *S*(*q*) (**a**), momentum distribution function *n*(*q*) (**b**), and static density response function $\chi (q)$ (**c**), see Eq. (1). Solid yellow: experimental data for *S*(*q*) by Hallock^47^.

In Fig. 2, we study the temperature dependence of various structural properties of normal liquid $^3{\text {He}}$, for $T=20, 5, 4, 3,$ and 2 K. Panel (a) shows $S({\mathbf {q}})$ that exhibits a weak dependence on *T* for $T\in [2,5]$. Here, the most pronounced temperature effect manifests in the long wavelength limit, which is determined by the isothermal compressibility^22^. We note that the exact $q\rightarrow 0$ limit cannot be accessed in our simulations due to the finite simulation cell^45,46^. Apart from this momentum quantization effect^45^, we find no finite-size effects in our PIMC results; see the “Methods” section for a corresponding analysis. Both the position and the shape of the peak are hardly affected by *T* in this regime, which is consistent with earlier findings for $^4{\text {He}}$^22^. The solid yellow line in panel (a) shows experimental data for $S({\mathbf {q}})$ at $T=0.41\;{\text {K}}$ by Hallock^47^, which is in excellent agreement with our PIMC results for the lowest temperature. Finally, the grey triangles depict results for a substantially larger temperature of 20 K. These results are clearly distinct from the other curves, and we find temperature dependence in the small *q* regime and also around the peak, which decreases in magnitude and shifts to larger *q*.

Panel (b) shows the momentum distribution function $n({\mathbf {q}})$ that we have derived with the procedure described in Ref.^48^. For this property, the temperature plays an important role because the $^3{\text {He}}$ atoms are propelled to larger momenta by thermal excitations. This can be seen particularly well for $T=20\;{\text {K}}$, where *n*(0) is reduced by more than a factor of four compared to 2 and 3 K results. Furthermore, $n({\mathbf {q}})$ does not resemble a step function even for the lowest depicted temperature ($T=2\;{\text {K}}$) for these reasons. The reduced temperature, $\Theta =k_{{\text {B}}}T/E_{{\text {F}}}\approx 0.4$, is not quite low enough, which is exacerbated by the strong pair interaction.

Lastly, panel (c) shows the static density response function^49^, which we derived from the relation^50,51^,

1 $$\begin{aligned} \chi ({\mathbf {q}}) = -n\int _0^\beta {{\text {d}}}\tau \ F({\mathbf {q}},\tau ), \end{aligned}$$

with the definition of the imaginary-time correlation function

2 $$\begin{aligned} F({\mathbf {q}},\tau ) = \left\langle {\hat{n}}({\mathbf {q}},0) {\hat{n}}(-{\mathbf {q}},\tau )\right\rangle , \end{aligned}$$

where ${\hat{n}}({\mathbf {q}},\tau )$ is the density operator in Fourier space evaluated at $\tau \in [0,\beta ]$; see also Ref.^50^ for a generalization. We find that $\chi ({\mathbf {q}})$ exhibits an interesting, non-monotonous structure: (1) In the limits of large and small *q*, the response function only weakly depends on temperature for $T\in [2,5]\text {K}$. Further, $\chi ({\mathbf {q}})$ does not approach zero in the long-wavelength limit, as there is no perfect screening^52,53^ for helium because of the strong short-range interactions^43^. (2) The density response function exhibits a pronounced peak around $q\approx 1.8$ Å$^{-1}$, which corresponds to $q\approx 2.25q_{{\text {F}}}$ (where $q_{{\text {F}}}$ is the Fermi wave number^54^). This feature closely resembles recent results^55,56^ for the uniform electron gas at warm dense matter conditions^57^ at similar values of the reduced temperature $\Theta$ and reduced wavenumber $x=q/q_{{\text {F}}}$. Finally, the peak of the density response substantially depends on *T*. Specifically, the peak location is directly connected to the attractive minimum of the inter-atomic potential. Increasing the temperature reduces the correlation among the atoms, there is less of a collective behaviour, which manifests itself in a weaker density response. This trend is confirmed by $T=20$ K results. $\chi ({\mathbf {q}})$ is substantially reduced compared to the other temperatures, especially for $q\lesssim 3q_{{\text {F}}}$.

**Figure 3:**
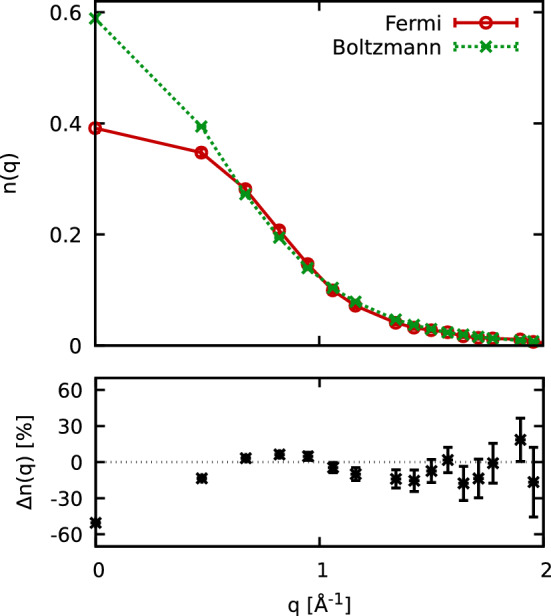
Impact of Fermi statistics on the momentum distribution *n*(*q*). Results from simulations of fermions and boltzmannons are compared for $T=2\;{\text {K}}$ in the upper panel and their relative deviations are shown below.

In Fig. 3, we study the impact of quantum statistics on the momentum distribution of $^3{\text {He}}$, $n({\mathbf {q}})$. For $T=2\;{\text {K}}$, we compare the results from our fermionic PIMC simulations (red) with the corresponding results for distinguishable particles, so-called *boltzmannons*. Evidently, the most pronounced differences appear for the zero-momentum state. Its occupation differs by approximately 50% because it is the momentum state with the highest occupation and therefore Pauli exclusion effects matter the most. Conversely, quantum statistical effect only change $S({\mathbf {q}})$ and $\chi ({\mathbf {q}})$ by little more than 1% at these conditions, which is consistent with previous findings for $^4{\text {He}}$^1^.

Finally we investigate is the dynamic structure factor $S({\mathbf {q}},\omega )$ of $^3{\text {He}}$, which we obtain by numerically inverting the equation^19^,

3 $$\begin{aligned} F({\mathbf {q}},\tau ) = \int _{-\infty }^\infty {{\text {d}}}\omega \ S({\mathbf {q}},\omega ) e^{-\tau \omega }. \end{aligned}$$

We employ a genetic algorithm similar to the scheme presented in Ref.^58^, which simultaneously minimizes the $\chi ^2$ measure of Eq. (3) as well as the first and inverse frequency moments; see the “Methods” section for more details. To our knowledge, no experimental measurements of $S({\mathbf {q}},\omega )$ exist in the temperature range $T\ge 2\text {K}$ that is accessible to our direct PIMC simulations. However, Sköld et al.^24^ presented neutron scattering data for $T=1.2$ K. Since our investigation of $S({\mathbf {q}})$ and $\chi ({\mathbf {q}})$, both of which are closely related to $S({\mathbf {q}},\omega )$, revealed no significant impact of quantum statistics, we have carried out PIMC simulations of $N=100$ $^3{\text {He}}$ atoms using Boltzmann statistics at this temperature.

**Figure 4:**
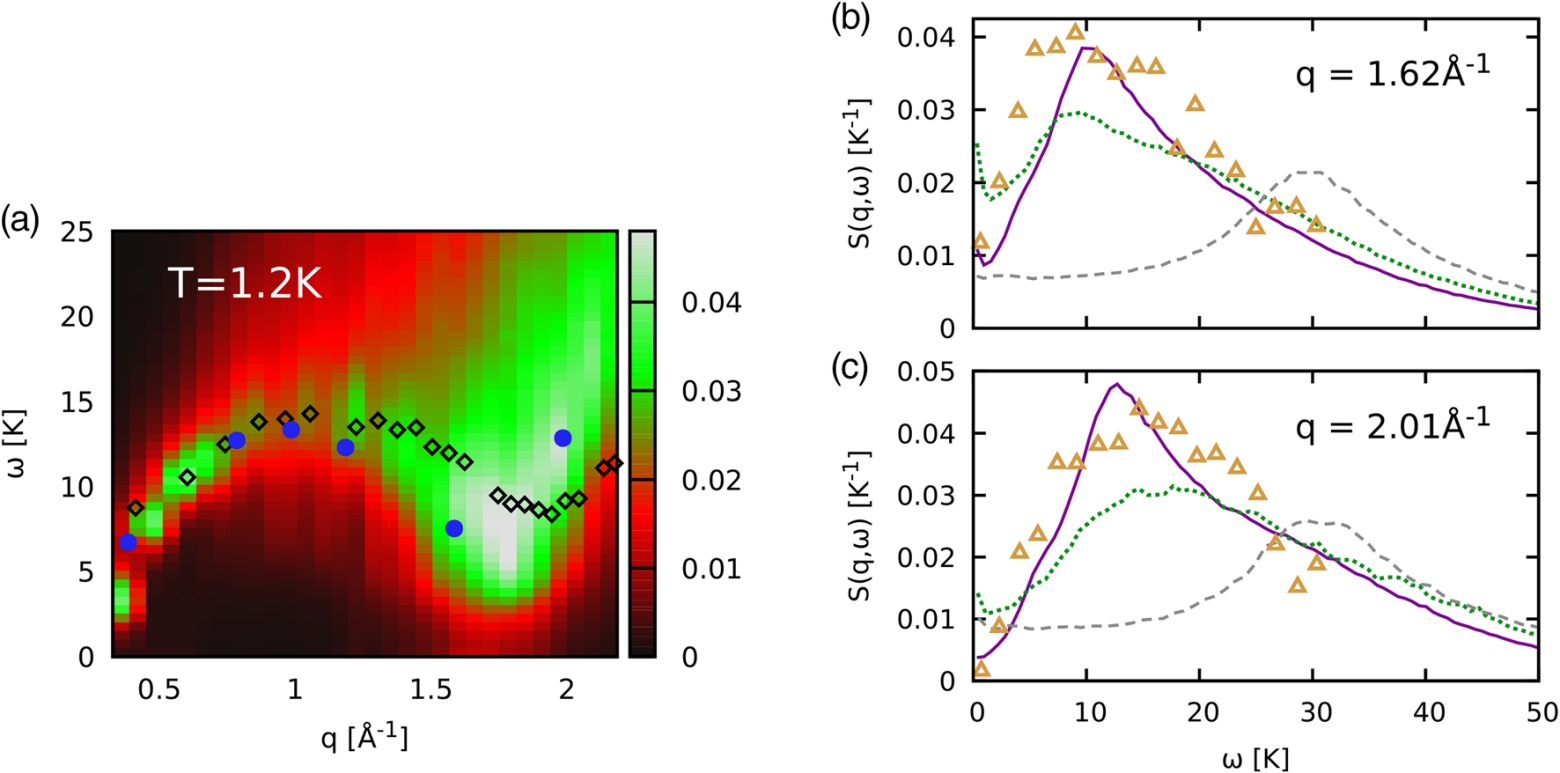
(**a**) Heatmap of reconstructed dispersion $S({\mathbf {q}},\omega )$ of $^3{\text {He}}$ at $T=1.2\;{\text {K}}$. Good agreement is found with the experimental data from Ref.^24^ (blue circles). Because of the higher mass, the roton minimum is shifted to higher *q* for $^4{\text {He}}$ as the theoretical results from Ref.^22^ show (black diamonds). (**b**, **c**) Dynamic structure factor $S({\mathbf {q}},\omega )$ for $q=1.62$ Å^−1^ and $q=2.01$ Å^−1^, solid purple: reconstructed PIMC data; yellow triangles: experimental data by Sköld et al.^24^.

The results of the numerical inversion of Eq. (3) are shown a heatmap in Fig. 4. All depicted datasets exhibit the phonon–maxon–roton dispersion relation that is well known for $^4\text {He}$. The blue circles are the experimental peak positions of $S({\mathbf {q}},\omega )$ from Ref.^24^. The experimental data are in excellent agreement to our results. Since we used Boltzmann statistics in our simulations, this is a strong indication that $S({\mathbf {q}},\omega )$ is predominantly shaped by the particle interactions rather than quantum statistical effects. Furthermore our results fully corroborate previous findings for $^3{\text {He}}$ in two dimensions^33,34^. The black diamonds show $S({\mathbf {q}},\omega )$ from a theoretical investigation of $^4{\text {He}}$ at the same *T*^22^ that differ substantially from our results for $q\gtrsim 1.3$ Å$^{-1}$. The differences between the two helium isotopes are mainly caused by the different particle masses because the heavier $^4{\text {He}}$ atoms are more strongly coupled than the $^3{\text {He}}$ species. Furthermore, our new PIMC results for $S({\mathbf {q}},\omega )$ clearly indicate the wave-number range for future neutron scattering experiments to resolve the precise location of the roton minimum in the spectrum.

Finally we investigate the $\omega$ dependence of $S({\mathbf {q}},\omega )$, which is shown in Fig. 4b,c for $q=1.62$ and 2.01 Å$^{-1}$. A comparison of the full $\omega$ dependence is of high importance to assess the quality of a theoretical method because the peak positions alone provide only limited information^59^. The yellow triangles depict the experimental measurements while the solid purple line shows the theoretical prediction from the analytic continuation. The experimental and theoretical dataset are in excellent agreement over the entire frequency range. This implies that the PIMC simulation and subsequent reconstruction are not only capable to reproduce the correct dispersion $\omega _{{\text {max}}}(q)$ but also gives access to the actual shape of $S({\mathbf {q}},\omega )$. From a physical perspective, we note that both considered wave numbers are located around the roton minimum (cf. Fig. 4), but do not exhibit the sharp quasi-particle excitation peak that is characteristic for the superfluid phase of $^4{\text {He}}$^22^.

To elucidate the temperature dependence of the dynamic structure factor, we include results for $T=3$ K (dotted green) and $T=20$ K (dashed grey) into panels (b) and (c) of Fig. 4. We find the same qualitative trend for both wave numbers, i.e., a broadening of the spectra with increasing temperature, and a shift of the maximum towards higher frequencies $\omega$ for $T=20\;{\text {K}}$. In addition, there appears a small positive feature around $\omega =0$ for $T=3~{\text {K}}$, which is particularly pronounced for $q=1.62$ Å$^{-1}$. We note that such a *diffusive feature* can be a consequence of the attractive minimum in the pair interaction^60–64^. On the other hand, the $\omega =0$ peak could easily be an artifact due to the ill-posed nature of the reconstruction of $S({\mathbf {q}},\omega )$ based on $F({\mathbf {q}},\tau )$. A more detailed investigation of this effect must include the combination of different methods and will be pursued in a future publication.

## Discussion

We have presented an extensive set of new PIMC results for normal liquid $^3{\text {He}}$. Specifically, we have carried out direct PIMC calculations over the temperature range from 2 to 20 K, which are computationally challenging because of the fermion sign problem, but are numerically exact within the given error bars. We computed rigorous theoretical results for different static properties of bulk $^3{\text {He}}$ like the static structure factor $S({\mathbf {q}})$, momentum distribution function $n({\mathbf {q}})$, and static density response $\chi ({\mathbf {q}})$. We found the correlation-induced peak of $\chi ({\mathbf {q}})$ to strongly depend on *T*, whereas $S({\mathbf {q}})$ remains almost unchanged over the 2–5 K temperature interval. The absence of nodal restrictions in our simulations allowed us unambiguously quantify the impact of Fermi statistics, which is pronounced for $n({\mathbf {q}})$ for small momenta, but practically negligible for $S({\mathbf {q}})$ and $\chi ({\mathbf {q}})$ at these temperature conditions.

Since quantum statistical effects change the latter quantities, we carried out PIMC simulations of $^3{\text {He}}$ using Boltzmann statistics at a lower temperature of 1.2 K because direct fermionic PIMC simulations would have been unfeasible due to the sign problem. This has allowed us to compare the dynamic structure factor $S({\mathbf {q}},\omega )$ to experimental measurements. The two sets of results were found to be in excellent agreement when the peak position $\omega _{{\text {max}}}(q)$ and the actual shape of the entire spectra are compared. We have found the familiar phonon–maxon–roton dispersion relation that is well known for $^4{\text {He}}$. This substantiates the previous findings for $^3{\text {He}}$ in two dimensions^33,34^, where it has been reported that the shape of the dispersion is predominantly shaped by the interaction and not by quantum statistical effects. In addition, we analyzed the temperature dependence of $S({\mathbf {q}},\omega )$. Primarily the spectra broaden with increasing temperature and we have found a possible diffusive feature at $T=3\;{\text {K}}$ that will be investigated in more detail in future works.

Overall, our new results considerably extend the current understanding of one of the most important and widely studied quantum systems in the literature, which is important in its own right. Our highly accurate results for different properties constitute a benchmark for other methods such as the fixed-node approximation, and may guide the development of new, computationally less expensive approximations. In addition, we have predicted the behaviour of $^3{\text {He}}$ at a number of temperatures, which can be checked in future experimental investigations. Possible extensions of our work might include simulations of quantum dipole systems and other types of fermionic ultracold atoms. This might provide additional insights into important physical effects like the formation of composite bosons in bilayer systems and indirect excitons^42^, and help to explicitly resolve the impact of quantum statistics on collective excitations^39,65^.

In principle, it is possible to study $^3{\text {He}}$ based on direct PIMC simulations in the grand-canonical ensemble^66^, which would give access to additional physical properties such as the compressibility and the single-particle spectrum $A({\mathbf {q}},\omega )$^65^.

Finally, we note that the direct PIMC simulation of 3He along the superfluid phase transition^32,67^ is presently not feasible because the fermion sign problem is too severe at the required temperatures but simulations of this transition remain an import open question for future investigations.

## Methods

### Verification of our PIMC implementation of helium

Accurate data for bulk $^3{\text {He}}$ at the conditions that we consider in the present work are sparse in the literature, which makes the verification of our numerical implementation with experimental data difficult. On the other hand, a gamut of theoretical works have been devoted to the bosonic simulation of $^4{\text {He}}$, which is typically simulated with the same pair potential. In addition, direct PIMC simulations of fermions may actually be derived from a reference systems of bosons. The (exact) information about the fermionic system is then extracted by taking into account the cancellation of positive and negative contributions due to the fermionic antisymmetry; see Refs.^29,68^ for details. Therefore, if we are able to obtain the correct permutation structure for $^4{\text {He}}$, we can verify the predictions of our direct PIMC simulations of fermionic $^3{\text {He}}$.

**Figure 5:**
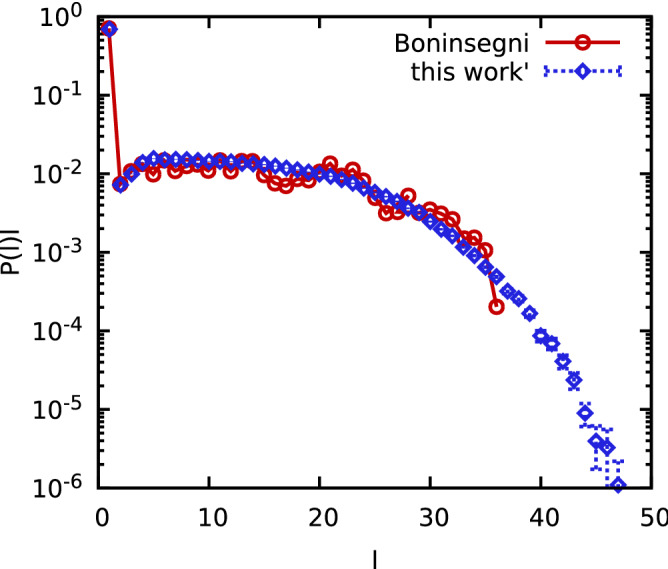
Comparing permutation-cycle probabilities for $^4\text {He}$ at $T=2\;{\text {K}}$ to previous results by Boninsegni^69^.

Therefore we have simulated $N=64$ spin-polarized $^4{\text {He}}$ atoms at $T=2$ K. The probability to find an individual particle involved in a permutation cycle of length *l*, *P*(*l*)*l*, is plotted in Fig. 5. The red circles show results from Boninsegni^69^ and the blue diamonds display the present work. First and foremost, we find excellent agreement between the two independent data sets over several orders of magnitude in the probability. In addition, we note that the curve from the present work is smoother compared to Ref.^69^ and includes results for longer permutation cycles, probably because we invested more computer time.

### Finite-size effects

**Figure 6:**
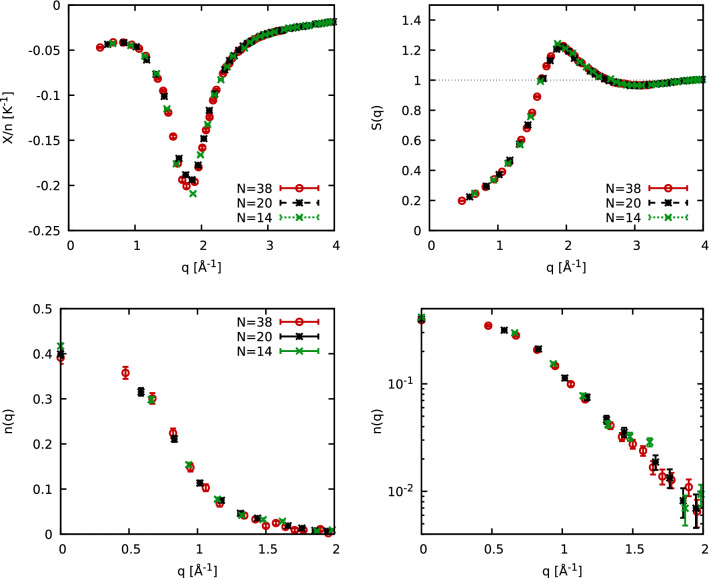
Finite-size effects in PIMC simulations of $^3{\text {He}}$ at $T=2$ K for the static density response function $\chi ({\mathbf {q}})$ (top left), the static structure factor $S({\mathbf {q}})$ (top right), and the momentum distribution function $n({\mathbf {q}})$ (bottom row).

The direct PIMC simulations that we employ throughout this work are capable to give exact expectation values for a given combination of temperature *T*, volume *V*, and particle number *N*. On the other hand, we are interested in the properties of bulk $^3{\text {He}}$ in the thermodynamic limit ($N\rightarrow \infty$ where the number density $n=N/V$ is being kept constant.) While the comparison of PIMC results to experimental measurements for both $S({\mathbf {q}})$ and $S({\mathbf {q}},\omega )$ presented in the main text is convincing, we feel that an additional analysis of finite-size effects in our PIMC data is pertinent. To this end, we show PIMC results for different properties and different numbers of particles in Fig. 6. The top left panel shows results for the static density response function $\chi ({\mathbf {q}})$. The red circles, black stars, and green crosses have been obtained for $N=38$, $N=20$, and $N=14$ $^3{\text {He}}$ atoms, respectively. For completeness, we note that we take into account the pair interaction between atoms both within the original simulation cell, and the $N_\mathbf{I }=3^3-1$ nearest images, which has a noticeable impact for the simulations with $N=14$ particles despite the relatively short-range nature of the employed Aziz potential^43^. Still all three datasets are in remarkably good agreement. No dependence on the system size can be resolved both for small and large *q*, and the small deviations between the black stars and green crosses around the peak in $\chi ({\mathbf {q}})$ do not exceed a few per cent.

The top right panel of Fig. 6 shows the corresponding results for the static structure factor $S({\mathbf {q}})$. The finite-size effects are found to be even smaller. Finally, the bottom row shows results for the momentum distribution function $n({\mathbf {q}})$ both on a linear (left) and logarithmic (right) scale. The linear scale allows us to analyse the behaviour at small momenta, whereas the logarithmic plot is well suited to resolve effects at large *q*. Yet, no significant deviations can be resolved between the three datasets in either panel.

### Convergence with the number of imaginary-time slices

An additional practical issue in PIMC simulations of either fermions, bosons, or boltzmannons is the convergence with the number of steps in imaginary time, *P*. In real space, the matrix elements of the density operator, ${\hat{\rho }}=e^{-\beta {\hat{H}}}$, are given by,

4 $$\begin{aligned} \rho ({\mathbf {R}},{\mathbf {R}}',\beta ) = \left\langle {\mathbf {R}}|e^{-\beta {\hat{H}}}|{\mathbf {R}}'\right\rangle , \end{aligned}$$

where ${\mathbf {R}}=({\mathbf {r}}_1,\ldots ,{\mathbf {r}}_N)^T$ contains the coordinates of all *N* $^3{\text {He}}$ atoms. These matrix elements are not exactly known because the kinetic (${\hat{K}}$) and potential (${\hat{V}}$) contributions to the full Hamiltonian ${\hat{H}}$ do not commute, $e^{-\beta {\hat{H}}}\ne e^{-\beta {\hat{K}}}e^{-\beta {\hat{V}}}$. As a practical workaround, we employ the exact semi-group property of the density operator

5 $$\begin{aligned} e^{-\beta {\hat{H}}} = \prod _{\alpha =0}^{P-1} e^{-\epsilon {\hat{H}}}, \end{aligned}$$

where $\epsilon =\beta /P$ is the time step of the path integral. Ultimately, Eq. (5) implies that we have to evaluate *P* density matrices of a temperature that is *P* times higher than the original one. This allows one to introduce a suitable high-temperature approximation, which becomes exact in the limit of large *P*.

In practice, we employ the *primitive approximation*

6 $$\begin{aligned} e^{-\epsilon {\hat{H}}} \approx e^{-\epsilon {\hat{K}}} e^{-\epsilon {\hat{V}}}, \end{aligned}$$

and the associated factorization error decays as $P^{-2}$; see Refs.^70,71^ for more detailed information on this point.

**Figure 7:**
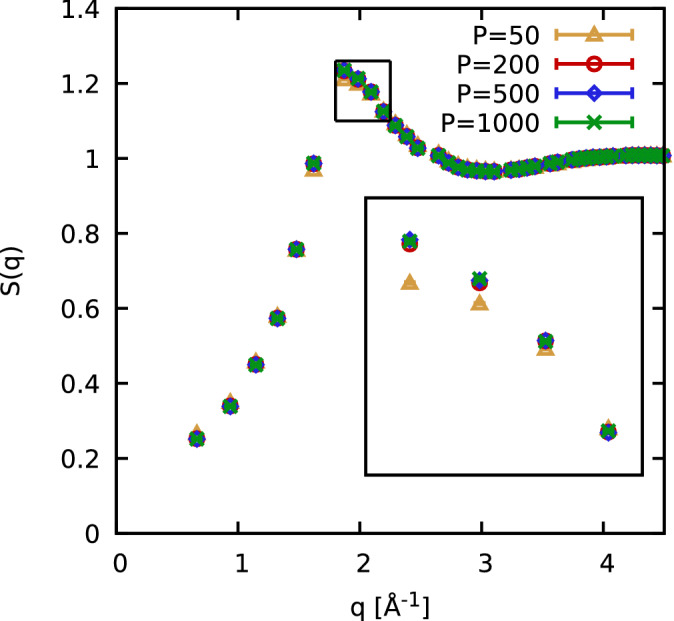
Convergence with the number of imaginary-time slices *P*. Shown are PIMC data for the static structure factor $S({\mathbf {q}})$ for $N=14$ $^3{\text {He}}$ atoms at $T=2.5\;{\text {K}}$.

As a practical example for the convergence with time step number *P*, we show results for the static structure factor $S({\mathbf {q}})$ for $N=14$ and $T=2.5\;{\text {K}}$ in Fig. 7. This observable constitutes a representative example, as it is directly connected to the dynamic structure factor $S({\mathbf {q}},\omega )$ [cf. Eq. (10)], to the imaginary-time correlation function $F({\mathbf {q}},\tau )$ by the relation $S({\mathbf {q}})=F({\mathbf {q}},0)$, and indirectly also to the static density response function $\chi ({\mathbf {q}})$, cf. Eq. (1). Evidently, a pronounced factorization error can only be resolved for $P=50$, while the results that were obtained with the higher *P* values are in very good agreement. $P=50$ is clearly not sufficient for the description of a strongly correlated quantum liquid. We used $P=1000$ throughout this work. Therefore, any residual factorization error are much smaller than the Monte Carlo error bars, and our results are thus well converged.

### Analytic continuation

The goal for the analytic continuation^19^ is to find a suitable trial solution for the dynamic structure factor $S_{{\text {trial}}}({\mathbf {q}},\omega )$ that, when it is being inserted into Eq. (3) of the main text, reproduces the PIMC data for the imaginary-time correlation function $F({\mathbf {q}},\tau )$ for all $\tau \in [0,\beta ]$. Yet, these constraints are typically not sufficient to fully constrain the space of possible solutions of $S({\mathbf {q}},\omega )$, and additional information are needed^39,72^. For this purpose, we consider the frequency moments of the dynamic structure factor, which are defined as

7 $$\begin{aligned} \left\langle \omega ^\nu \right\rangle = \int _{-\infty }^\infty {{\text {d}}}\omega \ S({\mathbf {q}},\omega ) \omega ^\nu . \end{aligned}$$

Two moments are known from the corresponding sum rules: (1) the first moment is determined by the well-known f-sum rule^54^

8 $$\begin{aligned} \left\langle \omega ^1\right\rangle = \frac{\hbar {\mathbf {q}}^2}{2m}, \end{aligned}$$

and the inverse moment is given by^73^

9 $$\begin{aligned} \left\langle \omega ^{-1}\right\rangle = -\frac{\chi ({\mathbf {q}})}{2n}. \end{aligned}$$

In addition, the zero-moment is automatically satisfied, since it holds

10 $$\begin{aligned} F({\mathbf {q}},0) = S({\mathbf {q}}) = \int _{-\infty }^\infty {{\text {d}}}\omega \ S({\mathbf {q}},\omega ) = \left\langle \omega ^0\right\rangle . \end{aligned}$$

The final exact property of $S({\mathbf {q}},\omega )$ that we consider in this work is the detailed balance relation between positive and negative frequencies^74^,

11 $$\begin{aligned} S({\mathbf {q}},-\omega ) = e^{-\beta \omega }S({\mathbf {q}},\omega ), \end{aligned}$$

which is automatically fulfilled by our trial solutions $S_{{\text {trial}}}({\mathbf {q}},\omega )$.

Many practical approaches have been suggested to accomplish goals of the analytic continuation. One family of methods is based on Bayes’ theorem, and is capable of producing smooth solutions without any unphysical sawtooth instabilities^19^. Yet, such *maximum entropy methods*^21^ might introduce an artificial a-priori bias into the solutions, although notable progress is continually being made in this area^20,75^. A second paradigm for the analytic continuation is based on the averaging over $N_{{\text {S}}}\sim 10^3$–$10^4$ noisy, independent trial solutions to compose a smooth result^22,58,65^. While computationally more demanding, this method has the advantage that unexpected physical features of $S({\mathbf {q}},\omega )$ might still be recovered because no explicit bias is introduced into the solution.

In the present work, we pursue the latter strategy and employ a genetic algorithm that maximizes the fitness function,

12 $$\begin{aligned} \Phi= & {} \left\{ \frac{1}{P/2+1}\sum _{\alpha =0}^{P/2}\frac{|F({\mathbf {q}}, \alpha \epsilon )-F_{{\text {trial}}}({\mathbf {q}},\alpha \epsilon )|}{\Delta F({\mathbf {q}},\alpha \epsilon )} + a_\chi \frac{|\chi ({\mathbf {q}})-\chi _{{\text {trial}}}({\mathbf {q}})|}{\Delta \chi ({\mathbf {q}})} + a_1 \frac{|\left\langle \omega ^1\right\rangle -\left\langle \omega ^1\right\rangle _{{\text {trial}}}|}{\Delta \left\langle \omega ^1\right\rangle } \right\} ^{-1}. \end{aligned}$$

We note that $F({\mathbf {q}},\tau )$ is symmetric around $\beta /2$ so that we only have to consider the interval $\tau \in [0,\beta /2]$. Furthermore, the weights $a_\chi$ and $a_1$ control the respective influence of the individual constraints and are chosen empirically. We find that a reasonable choice is given by $a_\chi = a_1 = 1/2$, which means that the frequency moments are of the same importance as $F({\mathbf {q}},\tau )$. The f-sum rule, Eq. (8), is actually known exactly, and we empirically set $\Delta \left\langle \omega ^1\right\rangle =\left\langle \omega ^1\right\rangle \times 10^{-3}$.

**Figure 8:**
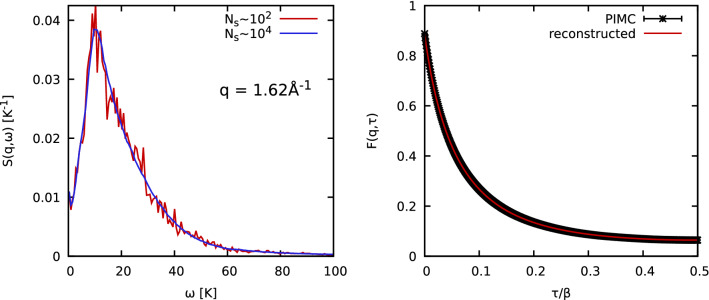
Left: Averaging of the dynamic structure factor $S({\mathbf {q}},\omega )$ for $T=1.2\;{\text {K}}$ and $q=1.62$ Å$^{-1}$. Red (blue) curve: average of $N_{{\text {s}}}\sim 10^2$ ($N_{{\text {s}}}\sim 10^4$) noisy individual solutions. Right: Comparing PIMC results for $F({\mathbf {q}},\tau )$ (black) and the reconstructed solution (red) for the same case as in Fig. 8. Note that $F({\mathbf {q}},\tau )$ is symmetric with respect to $\beta /2$, i.e., $F({\mathbf {q}},\tau )=F({\mathbf {q}},\beta -\tau )$.

A practical example for the analytical continuation is shown in the left panel of Fig. 8 for the case considered in Fig. 4b of the main text. The red (blue) curve has been composed by averaging over $N_{{\text {s}}}\sim 10^2$ ($N_{{\text {s}}}\sim 10^4$) individual noisy trial solutions $S_{{{\text {trial}}},i}({\mathbf {q}},\omega )$. A frequency grid with $\delta \omega \approx 0.6\;{\text {K}}$ and $N_\omega =400$ points was employed.

To conclude this discussion, we consider the main input of the analytic continuation method: the $\tau$-dependence of the imaginary-time correlation function $F({\mathbf {q}},\tau )$ for a specific wave number. This is shown for the present example in the right panel of Fig. 8, with the black stars and red line corresponding to the original PIMC results and the reconstructed solution, respectively. Evidently, the latter curve perfectly agrees with the PIMC data within the given Monte Carlo error bars that are on the order of $\Delta F\sim 10^{-3}$.

All $S({\mathbf {q}},\omega )$ results in this work have been obtained with the procedure that we just described but a note of caution is warranted. Despite being defined as an average value, it is not straightforward to estimate the true uncertainty in the final result for the reconstructed solution for $S({\mathbf {q}},\omega )$. Rather, the space of possible trial solutions remains a property of a specific optimization method (genetic algorithm, simulated annealing, etc.) and might, or might not, include the true, physical curve with finite probability. Therefore, we abstain from using the variance of the trial solutions as an error bar, as they might underestimate the true uncertainties.

## References

[1] Ceperley DM (1995). Path integrals in the theory of condensed helium. Rev. Mod. Phys..

[2] Yukalov VI (2011). Basics of Bose–Einstein condensation. Phys. Particles Nuclei.

[3] Feynman R, Hibbs A, Styer D (2010). Quantum Mechanics and Path Integrals. Dover Books on Physics.

[4] Chandler D, Wolynes PG (1981). Exploiting the isomorphism between quantum theory and classical statistical mechanics of polyatomic fluids. J. Chem. Phys..

[5] Fosdick LD, Jordan HF (1966). Path-integral calculation of the two-particle slater sum for $$\text{ he}^{4}$$. Phys. Rev..

[6] Herman MF, Bruskin EJ, Berne BJ (1982). On path integral Monte Carlo simulations. J. Chem. Phys..

[7] Takahashi M, Imada M (1984). Monte Carlo calculation of quantum systems. J. Phys. Soc. Jpn..

[8] Pollock EL, Ceperley DM (1984). Simulation of quantum many-body systems by path-integral methods. Phys. Rev. B.

[9] Foulkes WMC, Mitas L, Needs RJ, Rajagopal G (2001). Quantum Monte Carlo simulations of solids. Rev. Mod. Phys..

[10] Kolorenč J, Mitas L (2011). Applications of quantum Monte Carlo methods in condensed systems. Rep. Prog. Phys..

[11] Idrissi S, Labrim H, Ziti S, Bahmad L (2020). Characterization of the equiatomic quaternary heusler alloy zncdrhmn: Structural, electronic, and magnetic properties. J. Superconduct. Novel Magnet..

[12] Idrissi S, Labrim H, Ziti S, Bahmad L (2020). Structural, electronic, magnetic properties and critical behavior of the equiatomic quaternary heusler alloy cofetisn. Phys. Lett. A.

[13] Idrissi S, Labrim H, Ziti S, Bahmad L (2020). Investigation of the physical properties of the equiatomic quaternary heusler alloy coycrz (z = si and ge): A dft study. Appl. Phys. A.

[14] Idrissi S, Ziti S, Labrim H, Bahmad L (2020). Critical magnetic behavior of the rare earth-based alloy GDN: Monte Carlo simulations and density functional theory method. J. Mater. Eng. Perform..

[15] Dupé B, Kruse CN, Dornheim T, Heinze S (2016). How to reveal metastable skyrmionic spin structures by spin-polarized scanning tunneling microscopy. New J. Phys..

[16] Boninsegni M, Prokofev NV, Svistunov BV (2006). Worm algorithm and diagrammatic Monte Carlo: A new approach to continuous-space path integral Monte Carlo simulations. Phys. Rev. E.

[17] Boninsegni M, Prokofev NV, Svistunov BV (2006). Worm algorithm for continuous-space path integral Monte Carlo simulations. Phys. Rev. Lett..

[18] Pollock EL, Ceperley DM (1987). Path-integral computation of superfluid densities. Phys. Rev. Lett..

[19] Jarrell M, Gubernatis J (1996). Bayesian inference and the analytic continuation of imaginary-time quantum Monte Carlo data. Phys. Rep..

[20] Kora Y, Boninsegni M (2018). Dynamic structure factor of superfluid $$^{4}{\text{ He }}$$ from quantum Monte Carlo: Maximum entropy revisited. Phys. Rev. B.

[21] Boninsegni M, Ceperley DM (1996). Density fluctuations in liquid $$^{4}\text{ He }$$ path integrals and maximum entropy. J. Low Temp. Phys..

[22] Ferré G, Boronat J (2016). Dynamic structure factor of liquid $$^{4}\text{ He }$$ across the normal-superfluid transition. Phys. Rev. B.

[23] Yarnell JL, Arnold GP, Bendt PJ, Kerr EC (1959). Excitations in liquid helium: Neutron scattering measurements. Phys. Rev..

[24] Sköld K, Pelizzari CA, Mason R, Mitchell EWJ, White JW (1980). Elementary excitations in liquid $$^3$$He. Philos. Trans. R. Soc. Lond..

[25] Dietrich OW, Graf EH, Huang CH, Passell L (1972). Neutron scattering by rotons in liquid helium. Phys. Rev. A.

[26] Bramwell ST, Keimer B (2014). Neutron scattering from quantum condensed matter. Nat. Mater..

[27] Ceperley DM (1991). Fermion nodes. J. Stat. Phys..

[28] Troyer M, Wiese UJ (2005). Computational complexity and fundamental limitations to fermionic quantum Monte Carlo simulations. Phys. Rev. Lett.

[29] Dornheim T (2019). Fermion sign problem in path integral Monte Carlo simulations: Quantum dots, ultracold atoms, and warm dense matter. Phys. Rev. E.

[30] Greywall DS (1983). Specific heat of normal liquid $$^{3}{\text{ He }}$$. Phys. Rev. B.

[31] DuBois, J.L., Brown, E.W. & Alder, B.J. *Overcoming the Fermion Sign Problem in Homogeneous Systems*, chap. Chapter 13, 184–192. 10.1142/9789813209428_0013

[32] Vollhardt D, Wolfle P (2013). The Superfluid Phases of Helium 3. Dover Books on Physics Series.

[33] Godfrin H (2012). Observation of a roton collective mode in a two-dimensional fermi liquid. Nature.

[34] Nava M, Galli DE, Moroni S, Vitali E (2013). Dynamic structure factor for $${}^{3}$$He in two dimensions. Phys. Rev. B.

[35] Guckelsberger K (1989). Black is beautiful: $${}^{3}$$He—An experimental challenge to neutron spectroscopy. Physica B Condensed Matter.

[36] Boninsegni M, Ceperley DM (1995). Path integral Monte Carlo simulation of isotopic liquid helium mixtures. Phys. Rev. Lett..

[37] Boninsegni M, Moroni S (1997). Microscopic calculation of superfluidity and kinetic energies in isotopic liquid helium mixtures. Phys. Rev. Lett..

[38] Boninsegni M (2018). Kinetic energy and momentum distribution of isotopic liquid helium mixtures. J. Chem. Phys..

[39] Filinov A (2016). Correlation effects and collective excitations in bosonic bilayers: Role of quantum statistics, superfluidity, and the dimerization transition. Phys. Rev. A.

[40] Dornheim T (2020). Path-integral Monte Carlo simulations of quantum dipole systems in traps: Superfluidity, quantum statistics, and structural properties. Phys. Rev. A.

[41] Neumann M, Nyéki J, Cowan B, Saunders J (2007). Bilayer $${}^{3}$$He: A simple two-dimensional heavy-fermion system with quantum criticality. Science.

[42] Filinov A, Ludwig P, Bonitz M, Lozovik YE (2009). Effective interaction potential and superfluid–solid transition of spatially indirect excitons. J. Phys. A Math. Theor..

[43] Aziz RA, Nain VPS, Carley JS, Taylor WL, McConville GT (1979). An accurate intermolecular potential for helium. J. Chem. Phys..

[44] Krauth W (2006). Statistical Mechanics: Algorithms and Computations. Oxford Master Series in Physics.

[45] Dornheim T (2016). Ab initio quantum Monte Carlo simulation of the warm dense electron gas in the thermodynamic limit. Phys. Rev. Lett..

[46] Dornheim T, Groth S, Bonitz M (2017). Ab initio results for the static structure factor of the warm dense electron gas. Contrib. Plasma Phys.

[47] Hallock RB (1972). Liquid structure factor measurements on $$^3$$He. J. Low Temp. Phys..

[48] Dornheim T, Böhme M, Militzer B, Vorberger J (2021). Ab initio path integral Monte Carlo approach to the momentum distribution of the uniform electron gas at finite temperature without fixed nodes. Phys. Rev. B.

[49] Nolting W, Brewer WD (2009). Fundamentals of Many-Body Physics: Principles and Methods.

[50] Dornheim T, Moldabekov ZA, Vorberger J (2021). Nonlinear density response from imaginary-time correlation functions: Ab initio path integral Monte Carlo simulations of the warm dense electron gas. J. Chem. Phys..

[51] Bowen C, Sugiyama G, Alder BJ (1994). Static dielectric response of the electron gas. Phys. Rev. B.

[52] Dornheim T, Groth S, Bonitz M (2018). The uniform electron gas at warm dense matter conditions. Phys. Rep..

[53] Kugler AA (1970). Bounds for some equilibrium properties of an electron gas. Phys. Rev. A.

[54] Giuliani G, Vignale G (2008). Quantum Theory of the Electron Liquid.

[55] Dornheim T (2019). The static local field correction of the warm dense electron gas: An ab initio path integral Monte Carlo study and machine learning representation. J. Chem. Phys..

[56] Dornheim T, Sjostrom T, Tanaka S, Vorberger J (2020). Strongly coupled electron liquid: Ab initio path integral Monte Carlo simulations and dielectric theories. Phys. Rev. B.

[57] Bonitz M (2020). Ab initio simulation of warm dense matter. Phys. Plasmas.

[58] Vitali E, Rossi M, Reatto L, Galli DE (2010). Ab initio low-energy dynamics of superfluid and solid $$^{4}{{\text{ H }}}{{\text{ e }}}$$. Phys. Rev. B.

[59] Ramakrishna K, Cangi A, Dornheim T, Baczewski A, Vorberger J (2021). First-principles modeling of plasmons in aluminum under ambient and extreme conditions. Phys. Rev. B.

[60] Canales M, Padró JA (1997). Static and dynamic structure of liquid metals: Role of the different parts of the interaction potential. Phys. Rev. E.

[61] Canales M, Padró JA (1999). Dynamic properties of Lennard–Jones fluids and liquid metals. Phys. Rev. E.

[62] Choi Y, Murillo MS (2021). Influence of dissipation and effective interaction on the dense plasma dynamic structure factor. Phys. Rev. E.

[63] Moldabekov ZA (2018). Structural characteristics of strongly coupled ions in a dense quantum plasma. Phys. Rev. E.

[64] Moldabekov ZA (2019). Dynamical structure factor of strongly coupled ions in a dense quantum plasma. Phys. Rev. E.

[65] Filinov A, Bonitz M (2012). Collective and single-particle excitations in two-dimensional dipolar Bose gases. Phys. Rev. A.

[66] Dornheim T (2021). Fermion sign problem in path integral Monte Carlo simulations: Grand-canonical ensemble. J. Phys. A Math. Theor..

[67] Ma T, Wang S (2008). Superfluidity of helium-3. Physica A Stat. Mech. Appl..

[68] Dornheim T, Groth S, Filinov AV, Bonitz M (2019). Path integral Monte Carlo simulation of degenerate electrons: Permutation-cycle properties. J. Chem. Phys..

[69] Boninsegni M (2005). Permutation sampling in path integral Monte Carlo. J. Low Temp. Phys..

[70] Brualla L, Sakkos K, Boronat J, Casulleras J (2004). Higher order and infinite trotter-number extrapolations in path integral Monte Carlo. J. Chem. Phys..

[71] Sakkos K, Casulleras J, Boronat J (2009). High order chin actions in path integral Monte Carlo. J. Chem. Phys..

[72] Dornheim T, Groth S, Vorberger J, Bonitz M (2018). Ab initio path integral Monte Carlo results for the dynamic structure factor of correlated electrons: From the electron liquid to warm dense matter. Phys. Rev. Lett..

[73] Dalfovo F, Stringari S (1992). Static response function for longitudinal and transverse excitations in superfluid helium. Phys. Rev. B.

[74] Glenzer SH, Redmer R (2009). X-ray Thomson scattering in high energy density plasmas. Rev. Mod. Phys..

[75] Fuchs S, Pruschke T, Jarrell M (2010). Analytic continuation of quantum Monte Carlo data by stochastic analytical inference. Phys. Rev. E.

